# A set of EST-SNPs for map saturation and cultivar identification in melon

**DOI:** 10.1186/1471-2229-9-90

**Published:** 2009-07-15

**Authors:** Wim Deleu, Cristina Esteras, Cristina Roig, Mireia González-To, Iria Fernández-Silva, Daniel Gonzalez-Ibeas, José Blanca, Miguel A Aranda, Pere Arús, Fernando Nuez, Antonio J Monforte, Maria Belén Picó, Jordi Garcia-Mas

**Affiliations:** 1 IRTA, Centre de Recerca en Agrigenòmica CSIC-IRTA-UAB, Carretera de Cabrils Km 2, 08348 Cabrils (Barcelona), Spain; 2 COMAV-UPV, Institute for the Conservation and Breeding of Agricultural Biodiversity, Universidad Politécnica de Valencia, Camino de Vera s/n, 46022 Valencia, Spain; 3 Departamento de Biología del Estrés y Patología Vegetal, Centro de Edafología y Biología Aplicada del Segura (CEBAS)- CSIC, Apdo. correos 164, 30100 Espinardo (Murcia), Spain; 4 Instituto de Biología Molecular y Celular de Plantas (IBMCP) UPV-CSIC, Ciudad Politécnica de la Innovación Edificio 8E, Ingeniero Fausto Elio s/n, 46022 Valencia, Spain

## Abstract

**Background:**

There are few genomic tools available in melon (*Cucumis melo *L.), a member of the *Cucurbitaceae*, despite its importance as a crop. Among these tools, genetic maps have been constructed mainly using marker types such as simple sequence repeats (SSR), restriction fragment length polymorphisms (RFLP) and amplified fragment length polymorphisms (AFLP) in different mapping populations. There is a growing need for saturating the genetic map with single nucleotide polymorphisms (SNP), more amenable for high throughput analysis, especially if these markers are located in gene coding regions, to provide functional markers. Expressed sequence tags (ESTs) from melon are available in public databases, and resequencing ESTs or validating SNPs detected *in silico *are excellent ways to discover SNPs.

**Results:**

EST-based SNPs were discovered after resequencing ESTs between the parental lines of the PI 161375 (SC) × 'Piel de sapo' (PS) genetic map or using *in silico *SNP information from EST databases. In total 200 EST-based SNPs were mapped in the melon genetic map using a bin-mapping strategy, increasing the map density to 2.35 cM/marker. A subset of 45 SNPs was used to study variation in a panel of 48 melon accessions covering a wide range of the genetic diversity of the species. SNP analysis correctly reflected the genetic relationships compared with other marker systems, being able to distinguish all the accessions and cultivars.

**Conclusion:**

This is the first example of a genetic map in a cucurbit species that includes a major set of SNP markers discovered using ESTs. The PI 161375 × 'Piel de sapo' melon genetic map has around 700 markers, of which more than 500 are gene-based markers (SNP, RFLP and SSR). This genetic map will be a central tool for the construction of the melon physical map, the step prior to sequencing the complete genome. Using the set of SNP markers, it was possible to define the genetic relationships within a collection of forty-eight melon accessions as efficiently as with SSR markers, and these markers may also be useful for cultivar identification in Occidental melon varieties.

## Background

Single-nucleotide polymorphisms (SNPs) are the most frequent type of variation found in DNA [1] and are valuable markers for high-throughput genetic mapping, genetic variation studies and association mapping in crop plants. Several methods have been described for SNP discovery [2]: SNP mining from expressed sequence tag (EST) databases [3]; based on array hybridization [4] or amplicon resequencing [5]; from the complete sequence of a genome [6] and more recently, using high-throughput sequencing technologies [7]. The discovery of SNP markers based on transcribed regions has become a common application in plants because of the large number of ESTs available in databases, and EST-SNPs have been successfully mined from EST databases in non-model species such as Atlantic salmon [8], catfish [9], tomato [10] and white spruce [11].

Melon (*Cucumis melo *L.) is an important crop worldwide. It belongs to the *Cucurbitaceae *family, which also includes cucumber, watermelon, pumpkin and squash. The melon genome has an estimated size of 450 Mb [12] and is a diploid with a basic chromosome number of x = 12. In recent years research has been carried out to increase the genetic and genomic resources for this species, such as the sequencing of ESTs [13], the construction of a BAC library [14], the development of an oligo-based microarray [15] and the development of a collection of near isogenic lines (NILs) [16]. Genetic maps have also been reported for melon, but they have been constructed with different types of molecular markers and genetic backgrounds [17-21], making it difficult to transfer markers from one map to another. The aim of the International Cucurbit Genomics Initiative (ICuGI) [22], currently in progress, is to obtain a consensus genetic map by merging genetic maps available using a common set of SSRs as anchor markers.

A double haploid line (DHL) population from the cross between the Korean accession PI 161375 (SC) and the *inodorus *type 'Piel de sapo' T111 (PS) was the basis for the construction of a genetic map with 221 co-dominant, transferable RFLP and SSR markers [21]. New EST-derived SSR markers, added to this map using a bin-mapping strategy with only 14 mapping individuals, gave a new map with 296 markers distributed in 122 bins and a density of 4.2 cM/marker [21]. There is a need for saturating the SC × PS genetic map with more markers that are amenable for large-scale genotyping, as are SNPs. In a preliminary experiment with melon, amplicon resequencing of 34 ESTs in SC and PS was used for SNP discovery, obtaining a frequency of one SNP every 441 bp and one indel every 1,666 bp [23]. The availability of more than 34,000 melon ESTs from normalized cDNA libraries from different melon genotypes and tissues [13] is a valuable resource for the identification of SNPs to be added to the current genetic map.

Genetic markers can also be used for variability analysis studies. In melon, there have been several attempts to elucidate intraspecific relationships among melon germplasm, using isozyme [24], RFLP [25], RAPD [26], AFLP [27] and SSR [28] markers, with SSRs the preferred marker for fingerprinting and genetic variability analysis in melon [28]. Due to the absence of a known set of SNPs in the species, this marker has not been compared with other types for variability analysis. It would be of special interest to have a set of these markers for a high-throughput system to identify the germplasm used in breeding programs, mainly from *inodorus *and the *cantalupensis *melon types.

The objectives of this work were to increase the marker resolution in the melon genetic map, discovering EST-SNPs in a melon EST database, and to study the performance of a subset of EST-SNPs for variability analysis in a collection of melon accessions.

## Results and discussion

### SNP discovery

Two strategies were used to discover SNPs in melon. The first was based on producing amplicons from randomly selected melon ESTs and resequencing the parental lines of the melon genetic map PI 161375 (SC) × 'Piel de sapo' T111 (PS). Primers were designed from 223 melon ESTs (Table 1). After discarding primers that did not amplify a PCR product, amplicons that did not produce high quality sequences and monomorphic amplicons, 93 ESTs (56.3%) showed at least one polymorphism between SC and PS.

**Table 1:** Amplicons designed from ESTs for SNP discovery

	Amplicons	Failed	Monomorphic	Polymorphic	Polymorphic amplicons*	*In silico *SNP validation
Random ESTs	223	58	72	93	56.3%	

*in silico *pSNPs	269	41	69	159	69.7%	51.8%

*in silico *pSCHs	97	14	57	26	31.3%	21.3%

TOTAL	589	113	198	278	58.4%	

ESTs were selected at random or chosen because they contained pSNPs or pSCHs in the MELOGEN database. Columns show the number of amplicons that failed to amplify or gave bad quality sequences, and monomorphic and polymorphic amplicons between SC and PS. The percentages of polymorphic amplicons and *in silico *SNPs that were validated are shown in the last two columns. (*) Polymorphic amplicons rate was calculated without considering failed amplicons.

The second strategy was the validation of *in silico *SNPs from the ICuGI database [22]. Three hundred and sixty-six *in silico *SNPs found in the database were selected, belonging to two types of SNPs: pSNP and pSCH (Table 1; see methods). Primers were designed from 269 ESTs containing pSNP and 97 containing pSCHs. Putative *in silico *SNPs were validated in 51.8% and 21.3% of the amplicons for pSNPs and pSCHs, respectively. In some instances additional SNPs were detected in the sequenced regions, giving a slightly higher percentage of polymorphic amplicons (69.7% and 31.3% for pSNP and pSCH amplicons, respectively). From the ESTs reported by Gonzalez-Ibeas et al. [13], 47.3% were obtained from two accessions of the 'Piel de sapo' cultivar type (Pinyonet and PS), and the remainder from two genotypes, the C-35 cantaloupe accession (29.3%) and the pat81 *agrestis *accession (23.4%). The pSNPs and pSCHs were deduced from this set of EST sequences, with a high proportion found between pat81 and 'Piel de sapo', and SNPs experimentally validated after resequencing amplicons from PS and SC. SC belongs to the *agrestis *melon type as the accession pat81 but has a different origin, so, as expected not all the SNPs were conserved between SC and PS, giving a pSNP validation of 51.8%. On the other hand, only 21.3% of the pSCHs were validated, indicating that many may represent sequencing errors or mutations introduced during the cDNA synthesis procedure. The SNPs in a subset of amplicons containing *in silico *SNPs between 'Piel de Sapo' and pat81 were validated using different genotyping methods (see below) rather than resequencing in PS and SC.

A total of 368 amplicons (random and containing *in silico *SNPs) were resequenced in PS and SC and produced 177.5 kb of melon DNA, with 431 SNPs and 59 short indels, at an average of one SNP every 412 bp and one indel every 3.0 kb, (Table 2). This is in agreement with the values obtained in a previous small-scale experiment using the same two melon accessions, which gave one SNP every 441 bp and one indel every 1.6 kb [23]. SC and PS belong to the *agrestis *(*C. melo *ssp. *agrestis*) and *inodorus *(*C. melo *ssp. *melo*) melon groups, respectively, which are two of the more distant groups in the species [28]. This may explain the relatively high frequency of SNPs between the cultivars.

**Table 2:** Frequency of SNPs and indels found after resequencing EST-derived amplicons

Amplicons sequenced in SC and PS	Length sequenced (bp)	SNPs	bp per SNP	indels	bp per indel	Reference
368	177,518	431	411.9	59	3,008.8	this report

34	15,000	34	441.2	9	1,666.6	[23]

Data from a previous report using the same two melon parental lines is shown as a comparison.

### SNP detection

Various detection methods were used for genotyping the SNPs in each EST. A restriction site around the SNP position, different in the parental sequences, was used to develop a CAPS marker for 103 EST-SNPs. When more than one SNP was discovered in one amplicon, we selected the most suitable SNP for detection using CAPS. When no restriction enzyme was available to produce a CAPS marker, we used the SNaPshot SNP detection system. Seventy-seven EST-SNPs were genotyped with SNaPshot. For 14 ESTs, PS and SC gave a different amplicon size, so they could be genotyped as SCAR markers. Four EST-SNPs were genotyped using DNA sequencing and two were converted into dCAPS. The SNP detection method used for each mapped EST-SNP is shown in Additional file 1.

### SNP variability

Forty-five SNPs (see Additional file 2) were randomly chosen to study their variability in a set of melon accessions of worldwide cultivar and botanical types (see Additional File 3). The *inodorus *cultivars were overrepresented in order to assess whether SNPs between distant melon accessions (SC and PS) were also variable among more closely related genotypes.

All SNPs were polymorphic and the mean major allele frequency was 0.69 (Table 3). Only one SNP (AI_24-H05) had a rare allele (frequency = 0.08), whereas the frequencies of the two alleles were similar in 28 SNPs (major allele frequency < 0.65). Average gene diversity (He) was 0.4 (ranging from 0.14 to 0.5). Forty-three SNPs yielded He > 0.20, demonstrating that most of the chosen SNPs were highly informative, as found for SNPs in rye [29] but contrasting with crops such as soybean [30] and wheat [31] where SNPs yielding rare alleles are more frequent.

**Table 3:** Gene diversity indexes for SNP and SSR alleles using all, *inodorus *or genotypes described in a previous study [28]

Genotypes	Marker type	Major allele frequency	Ho	He	He range
all	SNP	0.69	0.10	0.40	0.14–0.50

*inodorus*	SNP	0.85*	0.07	0.15	0–0.50

group used in [28]	SNP	0.63	0.09	0.47	0.16–0.50

group used in [28]	SSR	0.47	0.14	0.64	0.51–0.83

Ho, observed heterozygosity; He expected heterozygosity. * Major allele frequency was only calculated for polymorphic SNPs.

The mean gene diversity index for SNPs was considerably lower than the values reported for SSRs in melon (e.g. PIC = 0.58 [21], He = 0.66 [28]). To ensure the difference was not due to sampling, gene diversity indexes were estimated using a subset of genotypes that had been included in a previous study with SSRs [28] (see Additional file 3). The differences in gene diversity were confirmed, demonstrating that they were intrinsic to the different marker type. SNPs are biallelic, implying that the He value can not exceed 0.5, whereas SSRs are multiallelic and so it can be higher. Haplotypes may yield higher gene diversity values than individual SNPs and provide more efficient application of SNP markers [29].

All *inodorus *genotypes could be distinguished with the set of SNPs, although polymorphism was notably reduced (Table 3). Fourteen SNPs were monomorphic and 18 were informative (minor allele frequency > 0.1). As most of the SNPs were discovered between the *agrestis *and *inodorus *cultivar and not within *inodorus*, we expected the SNP polymorphism within *inodorus *to be lower. Nevertheless, these results demonstrate that SNPs discovered using a germplasm sample can be successfully transferred to different germplasm samples in melon.

**Figure 1:**
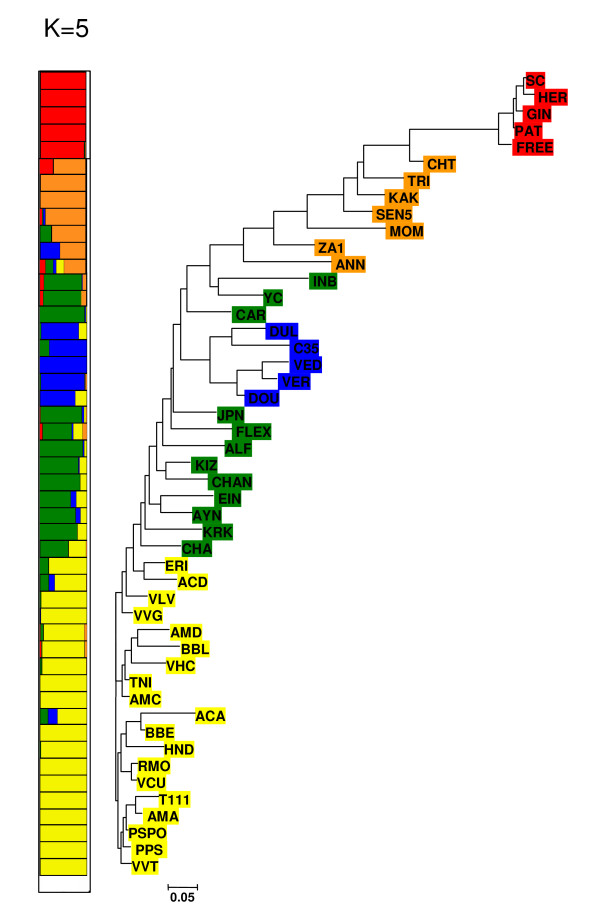
**Dendogram and population structure based on the variability of 45 SNPs in 48 melon accessions**. The neighbor-joining (NJ) tree based on Nei genetic distances [44] for the selected melon accessions is shown on the right. The subdivision based on STRUCTURE is shown on the left; each accession on the NJ is colored according to its group assignation defined from STRUCTURE analysis.

The genetic relationships among accessions based on SNP polymorphism were investigated by cluster analysis. The NJ dendrogram (Figure 1) fits very well with previous classifications using different markers [26,28,32]. Comparing the common genotype set in [28], the average pair-wise distances based on SNPs and SSR were 0.47 and 0.64, respectively. The correlation between the two distance matrices was 0.73 (P < 0.00001) according to Mantel's test, confirming that the current SNP set is as effective as SSRs in establishing genetic relationships among melon accessions, as shown in species such as rye [29] and soybean [30].

The population structure was estimated using the STRUCTURE software [33]. The *a posteriori *probability of the data increased rapidly from K = 1 to 4 and begun to reach a plateau for K = 5, inferring that our collection can be divided in five populations. Genetic variability among melon germplasm seems to be highly structured. The subdivision of the accessions in 5 populations agrees with the botanical classification and the cluster analysis (Figure 1): group 1 included all the *inodorus *cultivars from Spain; group 2, a diverse group of traditional *inodorus *landraces and similar ones from the Near-East region such as elongated (*chate *and *flexuosus*) and Asiatic *ananas *and *chandalak *types; group 3, modern *cantalupensis *cultivars; group 4, mainly traditional varieties and wild melons from India and Africa and group 5 included *conomon *accessions from the Far East. The population structure should be taken into account when establishing a collection of genotypes for association mapping studies in melon and models including population structure should be used [34]. Alternatively, melon collections without structure, as we found with the *inodorus *melon accessions included in our studies, could be used.

These results demonstrate that SNPs discovered using a small germplasm sample can be transferred to different cultivar groups, being useful for depicting genetic relationships as well as for cultivar identification.

### SNP mapping using a bin-mapping strategy

**Figure 2:**
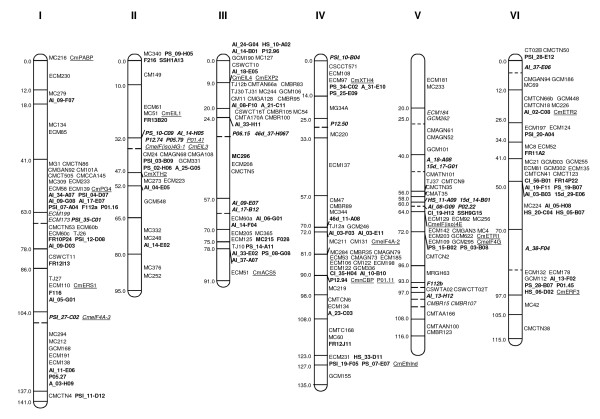
**EST-SNP bin map of *Cucumis melo *obtained by selective genotyping of fourteen DHLs**. Linkage groups are represented by vertical bars, divided in bins defined by the joint genotype of the selected DHLs. The mapped SNPs in this report are shown in bold. Underlined markers are candidate genes previously reported [23,35,36]. The other markers have been described in [21]. Genetic distances are shown on the left, indicating the position of the last marker included in the bin according to the framework map in [21]. Markers defining new bins are shown in italics. The hypothetical position of the last marker of these bins is indicated by a dashed horizontal line within the linkage group bar, without the genetic distance.

**Figure 3:**
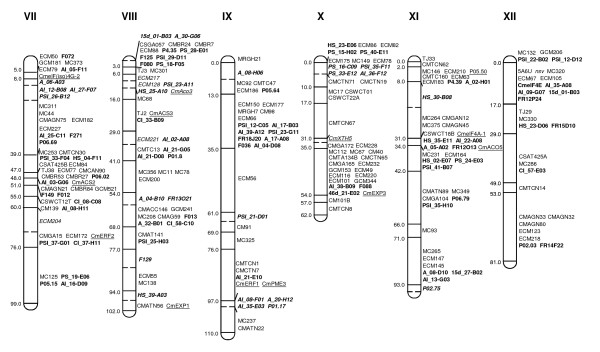
**EST-SNP bin map of *Cucumis melo *obtained by selective genotyping of fourteen DHLs**. Linkage groups are represented by vertical bars, divided in bins defined by the joint genotype of the selected DHLs. The mapped SNPs in this report are shown in bold. Underlined markers are candidate genes previously reported [23,35,36]. The other markers have been described in [21]. Genetic distances are shown on the left, indicating the position of the last marker included in the bin according to the framework map in [21]. Markers defining new bins are shown in italics. The hypothetical position of the last marker of these bins is indicated by a dashed horizontal line within the linkage group bar, without the genetic distance.

Two hundred and seventy-eight SNP-containing ESTs (Table 1) plus twelve additional SNP-containing ESTs previously discovered between the two parental lines [22] were used for mapping in the SC × PS genetic map using 14 DHLs of the melon bin-mapping population [21]. In total, 199 EST-derived SNPs were mapped, yielding 200 new markers (Figures 2 and 3). F112 produced two SCAR markers (F112a and F112b) that mapped to groups I and V, respectively. Our previous melon bin-map contained 296 markers distributed in 122 bins, with a density of 4.2 cM/marker and 2.4 markers per bin [21]. With the addition of 35 candidate genes previously reported for resistance to virus and fruit ripening [23,35,36] and the SNPs now described, the new bin-map contains 528 markers, distributed in 145 bins, with an increased density of 2.35 cM/marker and 3.64 markers per bin. The SNP-based markers defined 23 new bins with an average bin length of 8.55 cM. Some of the new bins were located in regions with poor marker density in the previous SC × PS melon map [21], such as HS_30-B08 in group XI, AI_12-B08 in group VII, A_38-F04 in group VI or P06.05 in group III.

Essentially the new version of the melon bin-map is a gene-based map, with 412 markers (78%) obtained from gene sequences. Additionally, 114 RFLPs derived from ESTs were previously mapped in an F2 population from the cross SC × PS [37], and their approximate position can also be plotted in the corresponding bin-map. As a large proportion of the markers are codominant and based on gene sequences, this makes this map a very useful tool for melon breeding and comparative analysis in cucurbit species.

With the advent of next generation sequencing technologies, SNP discovery has become more feasible in non-model crop species, allowing the discovery of thousands of SNPs in a single experiment [7]. In *Eucalyptus grandis *more than 23,000 SNPs were discovered using 454 sequencing technology, with a validation rate of 83% [38]. In melon, a preliminary analysis of 100,000 reads obtained after 454 sequencing of leaf cDNAs from SC and PS produced more than 1,000 SNPs (Garcia-Mas, unpublished). This indicates that the use of next generation sequencing technologies is the next step towards saturation of the melon genetic map.

## Conclusion

The set of 200 SNP markers discovered and mapped have increased the marker resolution of the melon genetic map by defining new bins. The genetic map contains more than 500 gene-based codominant markers (SNPs, RFLPs and SSRs), which can be used as anchor points with other genetic maps in this species. This genetic map is also a useful resource for comparative mapping in the *Cucurbitaceae*, for the construction of the melon physical map and for sequencing the melon genome. Additionally, the set of SNPs has proven to be as useful as microsatellites for studying genetic relationships in melon and for varietal identification.

## Methods

### Plant material and DNA extraction

The parent lines of the melon double haploid line (DHL) mapping population, PI 161375 'Songwan Charmi' (SC) and 'Piel de sapo' line T111 (PS), were used for SNP discovery [20]. Fourteen DHLs from the SC × PS segregating population were used to bin-map the SNP set [21]. The 48 melon genotypes selected for analysis with a subset of SNPs (see Additional file 3) were obtained from the germplasm collection maintained at COMAV (Valencia, Spain) and from a previous study of germplasm variability using SSRs [28]. DNA from all genotypes was extracted using a modified CTAB method [27]. DNA of the forty-eight melon accessions was extracted from leaves of five individuals per accession to take into account the genetic variability within heterogeneous accessions.

### SNP discovery and detection

SNPs were discovered using two different strategies. Firstly, random ESTs were selected from the International Cucurbit Genomics Initiative (ICuGI) webpage [22]. Primer pairs were designed from each EST using the Primer3 software [39] with an average length of 20 nucleotides, a melting temperature around 60°C and an expected PCR product of 500–700 bp. Genomic DNA from the parental lines of the melon mapping population was amplified with each primer pair as previously described [23]. Amplified fragments were purified with Sepharose columns and sequenced using the ABI Prism BigDye Terminator Cycle Sequencing kit (Applied Biosystems, Foster City, CA, USA) in an ABI Prism 3130 sequencer (Applied Biosystems, Foster City, CA, USA). Sequences were aligned and screened for polymorphism with the Bioedit software [40]. Putative SNP positions were visually verified on the sequence chromatogram, and the genomic sequences compared with the original EST sequence to identify any introns. In the second strategy, *in silico *SNPs previously identified [13] using EST2uni [41] were classified as i) pSNPs, corresponding to SNPs present in at least two EST sequences from the same genotype in a given contig and with the same base change and ii) pSCHs, corresponding to single nucleotide variations in sequence that did not follow the above criteria for pSNPs. Selected pSNPs and pSCHs were verified in most cases after resequencing the parental lines of the melon mapping population. For a small subset, the SNP was verified with an appropriate SNP detection method.

Bioedit software was used to generate restriction maps from sequences obtained from SC and PS. SNPs (or indels) showing differential restriction maps were used to develop cleaved amplified polymorphic sequence (CAPS) markers. When no differential restriction maps were available, the ABI Prism SNaPshot ddNTP Primer Extension Kit (Applied Biosystems) was used for SNP genotyping [23]. Markers F112, 46d_11-A08, FR12J11, 15d_17-G01, P01.45, PSI_26-B12, F012, PS_18-F05, PS_16-C09, F088, A_02-H11, AI_13-G03 and FR15D10 produced amplicons of different sizes in the parental lines, which were not sequenced and were genotyped as sequence characterized amplified region markers (SCARs) after electrophoresis in agarose gels or using a LI-COR IR2 sequencer (Li-Cor Inc, Lincoln, Nebraska, USA). Markers PSI_12-D08 and PSI_35-F11 were converted into dCAPS markers [42]. Markers F028, F149, F080 and PSI_25-B05 were genotyped using direct sequencing.

### SNP mapping

SNPs and indels were mapped by selective genotyping using the bin-mapping strategy [43], adapted for the melon mapping population [21]. Fourteen out of 72 DHLs from the melon mapping population were selected to obtain the maximum resolution with a minimum number of genotypes. SNPs and indels were placed in the bin map by visual inspection of the genotypes predicted by the markers and genotypes in the bin set.

### Genetic variability analysis

Forty-five SNPs from 44 amplicons (two SNPs were selected from F241) were chosen for genetic variability analysis. SNPs were genotyped as CAPS or by pyrosequencing as shown in Additional file 2. Thirty SNPs, described in Additional file 1, were used. Twelve SNPs that were not polymorphic between SC and PS were also included in the variability analysis, and the primers for each amplicon are provided in Additional file 2. The SNPs CmERF1, CmPm3 and CmXTH5 have been previously described [36].

Eight SNPs were genotyped by minisequencing the region surrounding the polymorphism (two SNPs were detected for F241 in the same reaction). Pyrosequencing was performed using a PSQ™ HS 96 system (Pyrosequencing AB, Uppsala, Sweden) following the manufacturers' instructions. Primers were designed with the Pyrosequencing™ Assay Design Software (Biotage AB, Uppsala, Sweden). One of the amplifying primers was 5' end labeled with biotin, allowing the immobilization of the fragment onto M-280 streptavidin coated Sepharose™ dynabeads (Dynal AS, Oslo, Norway). The genotyping primer was hence designed to anneal several nucleotides upstream of the SNP. After denaturation of the streptavidin-captured PCR fragments, the single stranded DNA fragments were released into the wells of the PSQ HS 96 plate. Pyrosequencing was performed using the PSQ HS SNP Reagent kit (Pyrosequencing AB, Uppsala, Sweden), and bioluminometric quantification of pyrophosphate (Ppi) released as a result of nucleotide incorporation during DNA synthesis was measured with the PSQ™ HS 96 system.

Allele frequencies, major allele frequency, gene diversity (measured as expected heterozygosity, He [44]), genetic distances and neighbor-joining (NJ) tree were calculated using Powermarker 3.25 [45]. The NJ tree was plotted with MEGA 3.0 [46]. Distance matrices were compared by the Mantel test [47].

The number of populations in our collection was deduced with the STRUCTURE software [33]. This package uses a Bayesian clustering approach to identify subpopulations and to assign individuals to these populations on the basis of their genotypes. Given a sample of individuals, K populations are assumed (where K may be unknown) and individuals are assigned to these populations. *A posteriori *probability for each K (Pr(K)) can be calculated, which is very small for K values lower than the appropriate value. Usually, the researcher fixes a minimum K (for example K = 1), recording Pr(K) after the analysis, and tests increasing Ks, plotting K against Pr(K). The final K is defined when Pr(K) reaches a plateau for higher K values. Consequently, in the current report, several number of populations (from K = 1 to 8) were tested with the software and the total number of populations was set when the probability reached a plateau for higher K.

## Authors' contributions

WD discovered and mapped the SNPs and performed the genotyping for the variability analysis. CE and MGT discovered and mapped SNPs. CR discovered SNPs. IFS mapped SNPs. DGI identified and selected *in silico *SNPs. JB carried out the bioinformatics analyses for *in silico *SNPs. AJM performed the variability analysis, coordinated the SNP mapping and participated in the drafting of the manuscript. MBP prepared DNAs for the melon accessions and participated in the genotyping for the variability analysis and in the drafting of the manuscript. JGM, PA, FN, MBP and MAA were involved in the conception of the study. JGM is the principal researcher of this work, supervised it and wrote the manuscript. All authors read and approved the final manuscript.

## Supplementary Material

Additional file 1 **SNPs markers mapped in the SC × PS genetic map**. Shown here, for each SNP marker: the EST and accession number from where it was obtained; best BlastX hit and E-value for each EST; amplicon primer sequences; SNP/indel position; SNP detection method; linkage group and BIN where the marker maps to. ^a^sequence available in [22] or  without accession number. ^b^SNPs published by Morales et al (2004). ^c^SNP position is provided when located in exons and referred to EST in first column. ^d^third primer was used for SNaPshot genotyping. Click here for file

Additional file 2 **SNP markers used for genotyping the melon accessions**. The EST from where the SNPs were discovered, the genotyping method (CAPS or pyrosequencing), linkage group where the marker maps to, and source of the marker are given. For unmapped SNP markers, the amplicon primer sequences are given. For SNP markers genotyped using pyrosequencing, forward, reverse and internal primers were used for genotyping. 5'bio: Forward or reverse primer was 5' labeled with biotine. ST1: Additional file 1. Click here for file

Additional file 3 **Forty-eight melon accessions that were examined in this study**. Plant assignation (or common name), code used in the current study, accession number from the respective gene banks, cultivar group, origin and seed bank donor (COMAV, Instituto de Conservación y Mejora de la Agrodiversidad Valenciana, Valencia, Spain; USDA/ARS/NCRPIS, North Central Regional Plant Introduction Station, Ames, IA, USA; IPK, Institute of Plant Genetics and Crop Plant Research, Gatersleben, Germany; INRA, Institute Nationale de la Recherche Agronomique, Montfavet, Avignon, France; Semillas Fitó SA, Barcelona, Spain; ARO, Agricultural Research Organization, Ramat Yishay, Israel) are specified for each genotype. Accessions marked with (*) were previously used by Monforte et al. (2003) for an SSR study. Click here for file
